# Differential Regulation of Anthocyanin Synthesis in Apple Peel under Different Sunlight Intensities

**DOI:** 10.3390/ijms20236060

**Published:** 2019-12-01

**Authors:** Weifeng Chen, Mengxia Zhang, Guojing Zhang, Pengmin Li, Fengwang Ma

**Affiliations:** State Key Laboratory of Crop Stress Biology for Arid Areas/Shaanxi Key Laboratory of Apple, College of Horticulture, Northwest A&F University, Yangling 712100, China; c4611475@nwsuaf.edu.cn (W.C.); mz458@cornell.edu (M.Z.); zhanggj1230@163.com (G.Z.); fwm64@nwsuaf.edu.cn (F.M.)

**Keywords:** anthocyanin synthesis, sunlight, UV-B, DFR, UFGT, apple

## Abstract

Sunlight radiation is a main environmental factor which affects anthocyanin synthesis. To clarify the regulatory mechanism of sunlight on the synthesis of anthocyanin in apple peel, bagged apples were exposed to diverse intensities of sunlight through different shading treatments. Under an increased solar ultraviolet-B (UV-B) light intensity, the concentration of anthocyanin in apple peels was consistent with the Michaelis–Menten equation. Under lower sunlight intensities, diphenyleneiodonium chloride (DPI, an inhibitor of plasma membrane NAD(P)H oxidase) treatment increased both the concentration of cyanidin-3-glycoside and the activity of dihydroflavonol 4-reductase (DFR). However, under higher sunlight intensities, DPI treatment decreased the concentrations of cyanidin-3-glycoside and quercetin-3-glycoside, as well as the activities of DFR and UDP-glycose: flavonoid 3-O-glycosyltransferase (UFGT). These results indicate that, under low sunlight intensity, anthocyanin synthesis in apple peel was limited by the supply of the substrate cyanidin, which was regulated by the DFR activity. Nevertheless, after exposure to high sunlight intensity, the anthocyanin produced in the apple peel was dependent on UFGT activity.

## 1. Introduction

Apples are an important agricultural crop and anthocyanins are the visible sign of apple maturity [1]. Anthocyanins belong to the reddish secondary metabolites, which consist of anthocyanidin and glycoside. They can increase the commerciality of various health foods and extend their shelf life [2,3]. Anthocyanins act as visual signals to attract insects for pollination and agents for seed dispersal [4], and also function as anti-oxidants, scavenging free radicals and protecting plant tissues against both biotic and abiotic stresses [5]. These compounds also have a positive effect on human health, serving as vasodilators and reducing the risk of myocardial infarction [6,7]. Anthocyanins have potential anti-diabetic properties; in addition, anthocyanin-rich foods can reduce starch consumption and delay glucose absorption by inhibiting alpha-amylase and alpha-glucosidase [8]. Anthocyanins can also benefit our bodies in terms of neuroprotection, vision improvement, anti-inflammatory effects, antimicrobial activity, chemoprevention, and cancer protection [9,10,11]. 

The synthesis of anthocyanins is involved in the phenylpropanoid metabolic pathway. Recently, the regulatory mechanisms of anthocyanin synthesis were reported [12,13,14,15,16,17,18]. Sunlight is one of the environmental factors regulating gene expression and plant development, and ultraviolet-B (UV-B) is considered to be a major factor to increase the synthesis of anthocyanins and flavonoids in plants [12,15]. UV-B induced photomorphogenesis is initiated by the specific photoreceptor, UV RESISTANCE LOCUS 8 (UVR8), which utilizes its tryptophan residue as an internal chromophore to sense UV-B [18]. UVR8 regulates the light signaling factors E3 ubiquitin ligase CONSTITUTIVELY PHOTOMORPHOGENIC1 (COP1) and bZIP transcription factor ELONGATED HYPOGOTYL5 (HY5) [18,19]. When COP1 and HY5 are combined with the MYB transcription factor, they regulate anthocyanin accumulation [20,21,22].

The biosynthesis of anthocyanins in apple skin mainly depends on the MYB-bHLH-WD40 (MBW) complex [23,24]. At low temperature and under artificial UV-B conditions, *MdbHLH3* binds to promoters of the anthocyanin synthesis genes *MdDFR* and *MdUFGT* to activate their expressions in apple fruit [25]. MYB10 regulates the synthesis of anthocyanins by regulating the activity of UDP-glycose: flavonoid 3-O-glycosyltransferase (UFGT) under partly-filtered solar UV irradiation conditions [12]. Previous apple studies have shown that chalcone synthase (CHS) is not the rate-limiting step in anthocyanin synthesis [26,27], but that its overexpression can significantly increase the accumulation of anthocyanins and upregulate the expression levels of *AtDFR* and *AtANS* under strong light in *Arabidopsis* [28]. Our previous studies demonstrated that chalcone isomerase (CHI) and flavanone 3-hydroxylase (F3H) were not key enzymes controlling the synthesis of anthocyanins and flavonols in apple [29]. Hydroflavonol 4-reductase (DFR) is a key enzyme in the biosynthesis of anthocyanins and has been extensively studied in many plants [30,31,32]. In apple peels, *CHS*, *F3H*, *DFR*, *ANS,* and *UFGT* achieved higher expression levels in UV-B and low-temperature conditions [33]. Previous studies have provided direct evidence that *MYBA* activates the *DFR* promoter in blueberry plants and the *ANS* promoter in apple peel [34,35]. The UV-B-specific perception and signaling pathways involving UVR8/COP1/HY5 have been suggested to work under narrow-band, low UV-B conditions, while the activation of reactive oxygen species (ROS)-mediated signaling, DNA damage response phytohormones, and mitogen-activated protein kinase (MAPK) signaling are promoted under broad-band, high UV-B conditions [36,37]. Through UV-light attenuation and diphenyleneiodonium chloride (DPI, an inhibitor of plasma membrane NADPH oxidase) treatment, it was found that the anthocyanin synthesis in ‘Golden Delicious’ was regulated by the ROS produced via plasma membrane NADPH oxidase [12]. Therefore, different UV-B irradiation conditions might regulate anthocyanin synthesis through different signaling pathways and structural genes.

In natural conditions, the radiation intensity of sunlight changes throughout the day. Due to shading of the tree canopy, each fruit receives a diverse intensity of UV-B radiation. With this variability, the structural genes which play important roles in the regulation of anthocyanin synthesis might also be different. In this study, we analyzed anthocyanin biosynthesis in both ‘Fuji’ and ‘Red Delicious’ apples, treated with diphenyleneiodonium chloride (DPI) which is an NAD(P)H oxidase inhibitor that can restrained ROS to affect anthocyanin synthesis, upon exposure to diverse sunlight intensities, in order to gain further insights into the regulation of anthocyanin synthesis.

## 2. Results

### 2.1. Anthocyanin Concentrations under Different Sunlight Intensities Fit with Michaelis–Menten Equation

**Figure 1:**
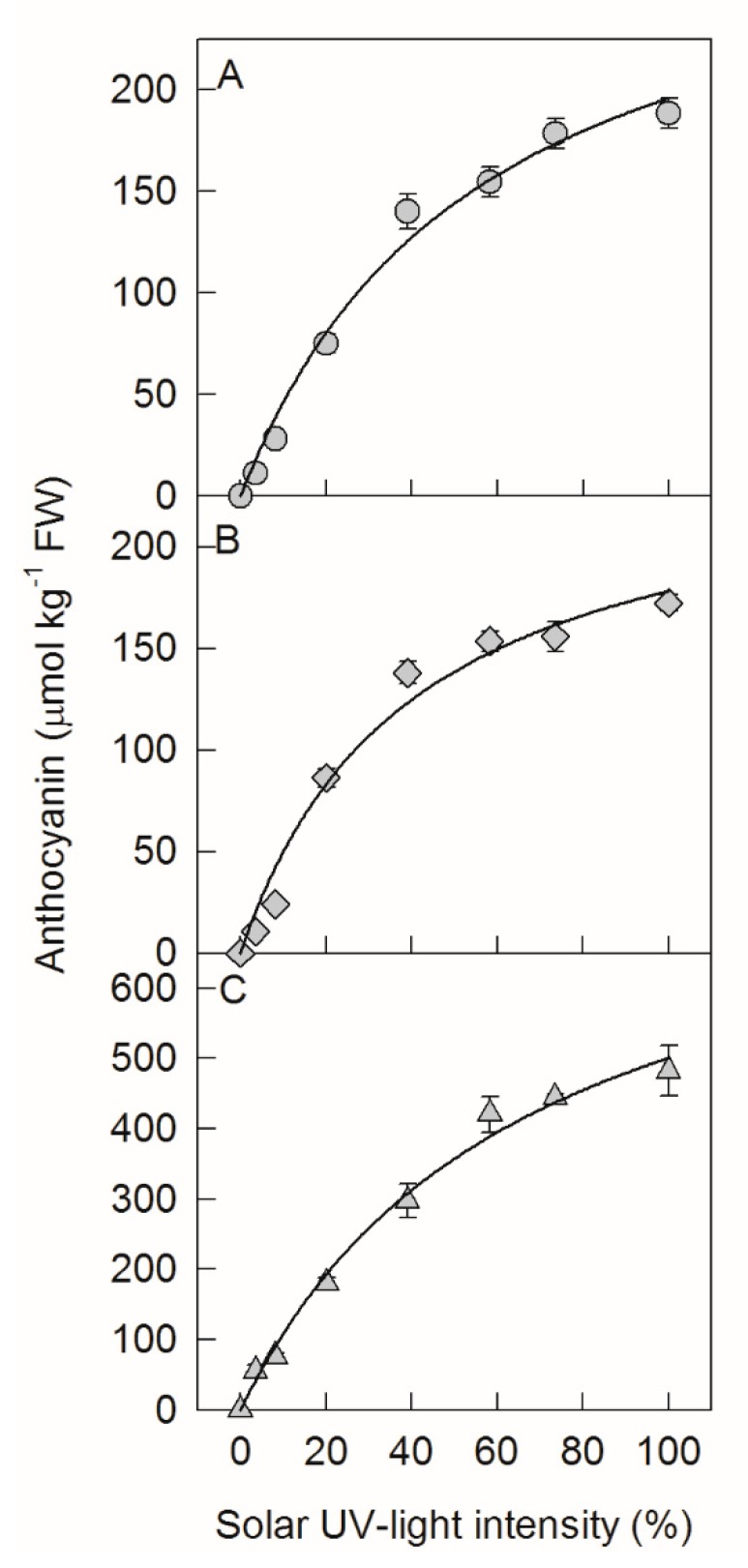
Concentrations of anthocyanin in ‘Fuji’ apple peel in 2016 (**A**) and 2017 (**B**) and ‘Red Delicious’ apple peel in 2017 (**C**) after exposing bagged fruits to diverse sunlight intensities. Each data point represents mean ± SE (*n* = 5). The fitting formula were y = $\frac{305.4x}{55.9+x}\;$, R^2^ = 0.988 (**A**), y = $\frac{249.6x}{40.1+x}\;$, R^2^ = 0.979 (**B**), and y = $\frac{836.9x}{67.1+x}\;$, R^2^ = 0.992 (**C**), respectively.

Through different shading treatments, the apple fruits were exposed to different intensities of sunlight. With increased solar UV-B light intensity, the concentration of total anthocyanins (cyanidin-3-galacotoside plus cyanidin-3-glucoside) in both ‘Fuji’ and ‘Red Delicious’ apple peels initially increased in a linear pattern, and then gradually leveled off in either 2016 or 2017 (Figure 1A–C). Moreover, the concentrations of anthocyanins under different light intensities showed a good fit with the Michaelis–Menten equation, at R^2^ values (the fitting formula) of 0.988, 0.979, and 0.992. 

### 2.2. Analysis of Anthocyanin and Flavonol Concentrations under Different Sunlight Intensities

**Figure 2:**
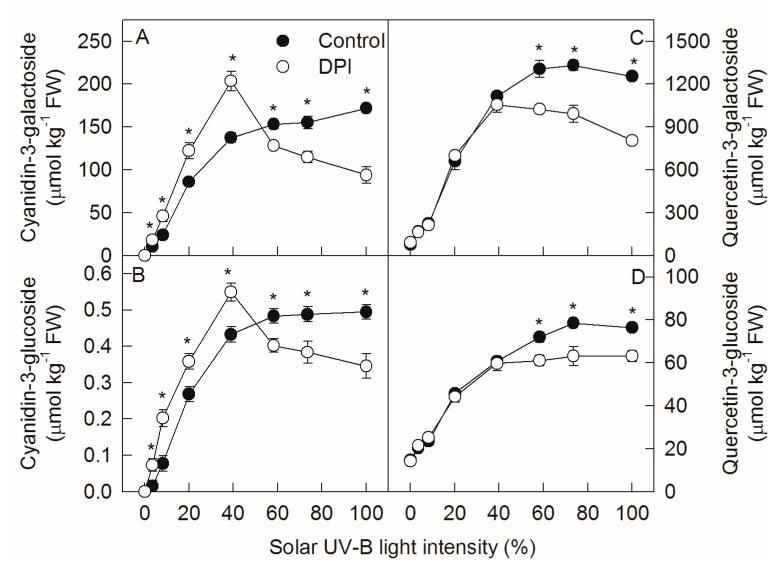
Concentrations of cyanidin-3-galactoside (**A**), cyanidin-3-glucoside (**B**), quercetin-3-galactoside (**C**), and quercetin-3-glucoside (**D**) in ‘Fuji’ apple peels after exposing bagged fruits, with or without diphenyleneiodonium chloride (DPI) treatment, to diverse sunlight intensities. Each data point represents mean ± SE (*n* = 5). The asterisk indicates a significant difference between DPI treatment and no DPI treatment at *p* < 0.05 (*t*-test).

The concentrations of flavonol, quercetin-3-galactoside, and quercetin-3-glucoside changed in similar patterns to cyanidin-3-glycoside in ‘Fuji’ apple peel as the solar UV-B light intensity increased (Figure 2C,D). When the light intensity was below 50%, DPI treatment increased the concentration of cyanidin-3-glycoside, but did not change the concentration of quercetin-3-glycoside in ‘Fuji’ apple peel. When the solar light intensity was over 50%, DPI treatment decreased the concentrations of both cyanidin-3-glycoside and quercetin-3-glycoside.

### 2.3. Analysis of Nucleotide Sugar Concentrations under Different Sunlight Intensities

**Figure 3:**
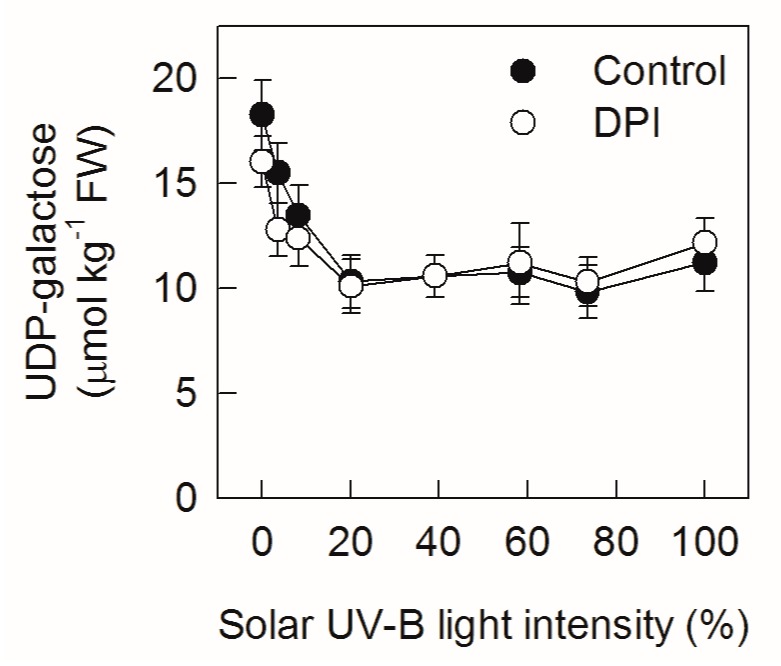
Concentrations of UDP-galactose in ‘Fuji’ apple peels after exposing bagged fruits, with or without DPI treatment, to diverse sunlight intensities. Each data point represents mean ± SE (*n* = 5).

As nucleotide sugar provides glycosyl group for anthocyanin synthesis, the concentration of UDP-galactose was also assayed. The concentration of UDP-galactose in ‘Fuji’ apple peel declined and then remained at a stable level along with the increasing of solar UV-B light intensity (Figure 3). Treatment with DPI did not change the concentration of UDP-galactose.

### 2.4. Analyze Gene Expression Levels under Different Sunlight Intensities

**Figure 4:**
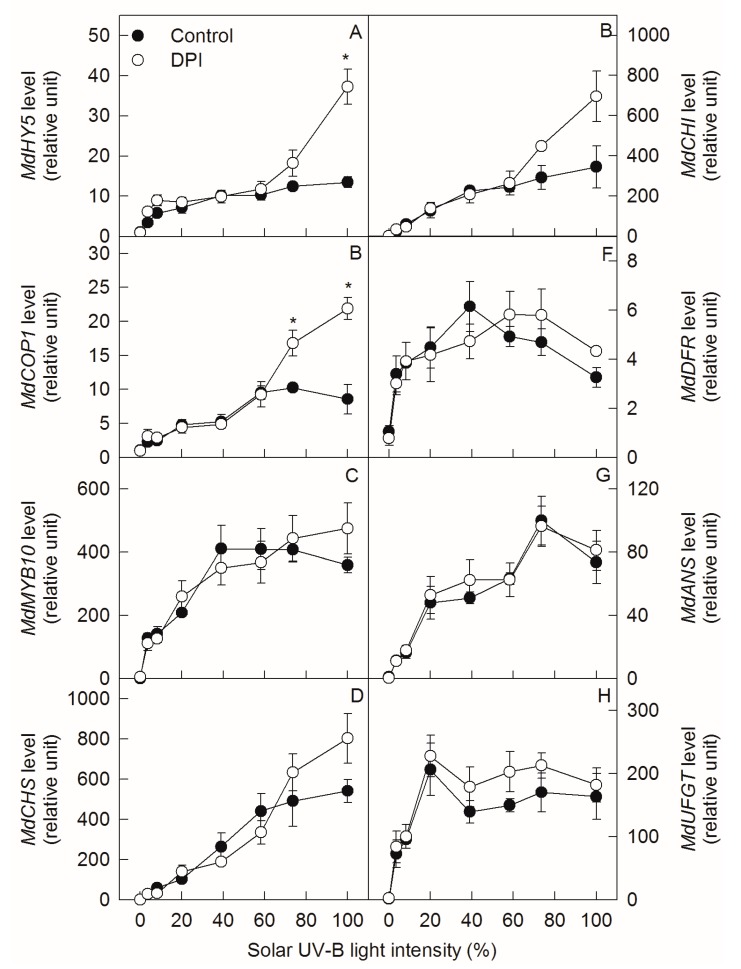
Transcription levels of *MdHY5* (**A**), *MdCOP1* (**B**), *MdMYB10* (**C**), *MdCHS* (**D**), *MdCHI* (**E**), *MdDFR* (**F**), *MdANS* (**G**), and *MdUFGT* (**H**) in ‘Fuji’ apple peels after exposing bagged fruits, with or without DPI treatment, to diverse sunlight intensities. Each data point represents mean ± SE (*n* = 5). The asterisk indicates a significant difference between DPI treatment and no DPI treatment at *p* < 0.05 (*t*-test).

Transcription levels of *MdUVR8*, *MdHY5*, *MdCOP1*, *MdMYB10*, *MdCHS*, *MdCHI*, *MdDFR, MdANS,* and *MdUFGT* in ‘Fuji’ apple peel initially increased and then remained unchanged with increasing solar UV-B light intensity, except for *MdDFR* and *MdUFGT,* which slightly decreased under higher light intensities (Figure 4A–H and Figure S1). DPI treatment did not change the gene levels, regardless of sunlight intensity, except for *MdHY5* and *MdCOP1,* which increased upon full exposure.

### 2.5. Analysis of Enzyme Activities under Different Sunlight Intensities

**Figure 5:**
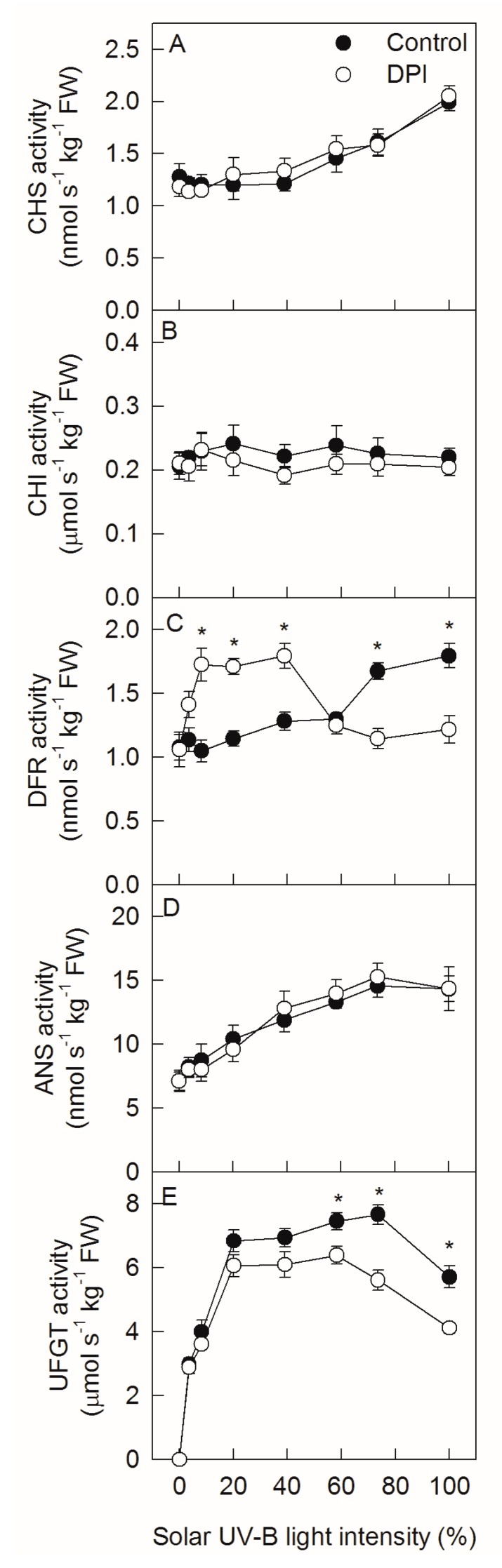
Activities of chalcone synthase (CHS, (**A**)), chalcone isomerase (CHI, (**B**)), dihydroflavonol 4-reductase (DFR, (**C**)), anthocyanidin synthase (ANS, (**D**)), and UDP-glycose: flavonoid 3-O-glycosyltransferase (UFGT, (**E**)) in ‘Fuji’ apple peels after exposing bagged fruits, with or without DPI treatment, to diverse sunlight intensities. Each data point represents mean ± SE (*n* = 5). The asterisk indicates a significant difference between DPI treatment and no DPI treatment at *p* < 0.05 (*t*-test).

The activities of CHS, DFR, and ANS in ‘Fuji’ apple peel continuously increased in accordance with increasing solar UV-B light intensity, but the CHI activity remained unchanged (Figure 5A–D). The UFGT activity increased linearly and remained steady with increasing intensity, but then slightly declined upon full exposure (Figure 5E). When the solar UV-B light intensity was below 50%, DPI treatment significantly increased DFR activity. When the solar UV-B light intensity was over 50%, DPI treatment decreased the activities of both DFR and UFGT. DPI treatment did not affect the activities of CHS, CHI, or ANS in ‘Fuji’ apple peel.

## 3. Discussion

In apple peel, the predominant anthocyanin component is cyanidin-3-galactoside [29,38,39]. The synthesis of cyanidin-3-galactoside was catalyzed by UFGT, namely, cyanidin + UDP-galactose $\overset{\mathrm{UFGT}}{\to }$ cyanidin-3-galacoside + UDP. The produced cyanidin-3-galacoside (A) is A = *V* × t, where *V* is the rate of cyanidin-3-galactoside synthesis, and t is time. According to the Michaelis–Menten equation, *V* = $\frac{Vmax\left[S\right]}{Km+\left[S\right]}$, and A = $\frac{Vmax\left[S\right]}{Km+\left[S\right]}$ × t. In this study, apples were exposed to different sunlight intensities for the same period of time. Thus, A at different light intensities was determined by *V*. Under low light intensity, *V*o = $\frac{Vmax\left[S\right]}{Km}\;$, and A = $\frac{Vmax\left[S\right]}{Km}$ × t. The produced cyanidin-3-galactoside depends on the concentration of the substrate [S]. Under high light intensity, *V*o = *V*max, namely, A = *V*max × t, and the produced cyanidin-3-galactoside depends on UFGT activity. Clearly, according to the Michaelis–Menten equation, the regulatory mechanism of anthocyanin synthesis in apple peel was different upon the exposure of bagged apples to diverse intensities of solar UV-B. When the solar UV-B light intensity was relatively low (below 50%), anthocyanin synthesis was limited by the supply of the substrate, whereas it was limited by UFGT activity when the solar UV-B light intensity was relatively high (over 50%, Figure 1 and Figure 5).

In our previous studies, after exposing bagged ‘Golden Delicious’ apples to sunlight, DPI (an inhibitor of plasma membrane oxidase) treatment inhibited the production of reactive oxygen species (ROS) via plasma membrane NADPH oxidase and the signaling pathway, reducing the expressions of *MdMYB10* and *MdUFGT,* the enzyme activity of UFGT, and the synthesis of anthocyanin in apple peel [12]. In this study, DPI treatment also inhibited anthocyanin synthesis in ‘Fuji’ apple peel when the bagged fruits were exposed to relatively high sunlight intensities (Figure 2). However, DPI did not affect the expression levels of *MdMYB10* and *MdUFGT*, but inhibited the enzyme activities of DFR and UFGT (Figure 4 and Figure 5). The different gene expression and enzyme activity responses might be related to the different cultivar properties, as ‘Golden Delicious’ is a non-red cultivar and ‘Fuji’ is a red cultivar. UFGT catalyzes the glycosylation of both cyanidin and quercetin in apple peel [27,40], while DFR is only involved in the synthesis of cyanidin [30,31,32]. Therefore, under relatively high sunlight conditions, the lower concentrations of quercetin-3-glycoside in DPI-treated ‘Fuji’ peels suggest that DPI may inhibit the synthesis of anthocyanin mainly by affecting the activity of UFGT (Figure 2). This is also consistent with the conclusion derived from the Michaelis–Menten equation, as mentioned above.

Interestingly, DPI treatment significantly increased the concentration of anthocyanin in ‘Fuji’ fruit peels after exposing the bagged fruits to relatively low sunlight conditions (Figure 2). This indicates that DPI treatment might have different effects on the regulation of anthocyanin synthesis in apple peel under diverse sunlight intensities. Indeed, DPI treatment did not inhibit the enzyme activity of UFGT, but increased that of DFR under relatively low sunlight conditions (Figure 5). DFR and ANS catalyze the synthesis of cyanidin, so the relatively higher DFR activity may provide more cyanidin for anthocyanin synthesis. According to the Michaelis–Menten equation, the synthesis of anthocyanin is limited by the substrate concentration under relatively low sunlight conditions (Figure 1). Since DPI treatment did not affect the concentration of UDP-Gal and the expression level and enzyme activity of ANS in apple peel (Figure 3, Figure 4G and Figure 5D), the supply of cyanidin catalyzed by DFR should be the limitation of anthocyanin synthesis under conditions of relatively low sunlight. The concentration of quercetin-3-glycoside was not affected by DPI treatment in relatively low sunlight conditions, indicating that other genes (enzymes) in the pathway shared by both anthocyanin and quercetin synthesis were not affected by DPI treatment. It is unclear why DPI treatment improved DFR activity under relatively low sunlight conditions. Wargent and Jordan (2013) suggested that anthocyanin synthesis is regulated through different signaling pathways under different UV-B irradiation conditions [37]. As anthocyanin was more sensitive to ROS than other flavonoid compounds in apple peel [41], it is perhaps that the more accumulated anthocyanins in DPI treated fruit peels were attributed to fewer ROS produced via plasma membrane NADPH oxidase. However, at first, under relatively low sunlight conditions, the produced ROS was also at low levels. Secondly, although ROS such as hydrogen peroxide may go through the cellular membrane, the enzymatic and non-enzymatic antioxidant systems in cytosol would be the first defense line against ROS prior to diffusion from the extracellular site of genesis to the vacuole where the anthocyanins are mainly located. In our previous studies, even at high light conditions, anthocyanin was not involved in the detoxification of ROS in pear peel [42]. 

**Figure 6:**
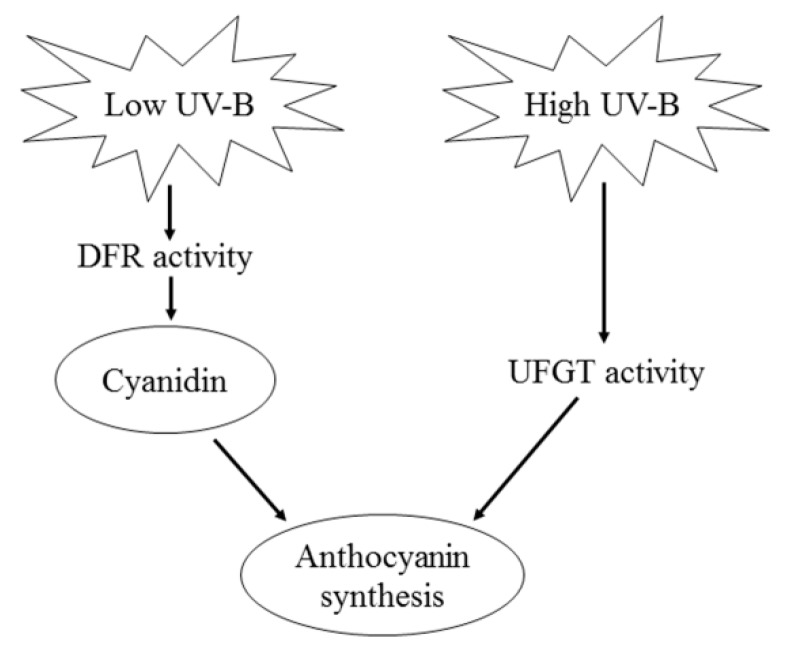
A regulation model of anthocyanin synthesis in apple peel under different sunlight intensities.

In conclusion, the regulatory mechanisms of anthocyanin synthesis in apple peel were different under diverse sunlight intensities. This might be the reason that previous studies have shown different limiting factors for anthocyanin synthesis in apple peel [12,20,34]. Under relatively low sunlight intensities, anthocyanin synthesis in apple peel was limited by the supply of the substrate cyanidin, which was regulated by the activity of DFR. However, under relatively high sunlight intensities, the produced anthocyanin is dependent on UFGT activity (Figure 6). These results might be useful for the development of new biotechnological strategies for improving the quality and market value of apples.

## 4. Materials and Methods

### 4.1. Plant Materials

Two apple cultivars (*Malus domestica* Borkh.), ‘Fuji’ and ‘Red Delicious’, were used in this study. ‘Fuji’ trees were field-grown at a spacing of 2.5 m × 3.5 m in Qianxian (34.53° N, 108.23° E; elevation 700 m), Shaanxi, China. ‘Red Delicious’ trees were field-grown at a spacing of 2.5 m × 3.5 m in Luochuan (35.76° N, 109.42° E; elevation 1033 m), Shaanxi, China. The plants were approximately 4 m tall with a central leader, and were grown using high quality horticultural cultivation techniques standard with disease and pest control. The apple fruits were bagged with light impermeable double-layer paper bags (the outer layer was colored yellow on the outside and black on the inside, the inner layer was red). The ‘Fuji’ fruits were bagged on 2 May 2016 and 3 May 2017 (approximately 30 days after full bloom), and were then harvested on 26 September 2016 and 27 September 2017 (approximately 176 days after full bloom), respectively. The ‘Red Delicious’ fruits were bagged on 10 May 2017 (approximately 30 days after full bloom), and were then harvested on 1 September 2017 (approximately 144 days after full bloom). Five replicates (ten fruits for each replicate) of the bagged fruits were sampled (three trees per replicate, 15 trees total) without removing the bags, to avoid exposure to light before chemical and sunlight exposure treatments.

### 4.2. Chemical and Diverse Intensities Solar UV-B Light Exposure Treatments

**Figure 7:**
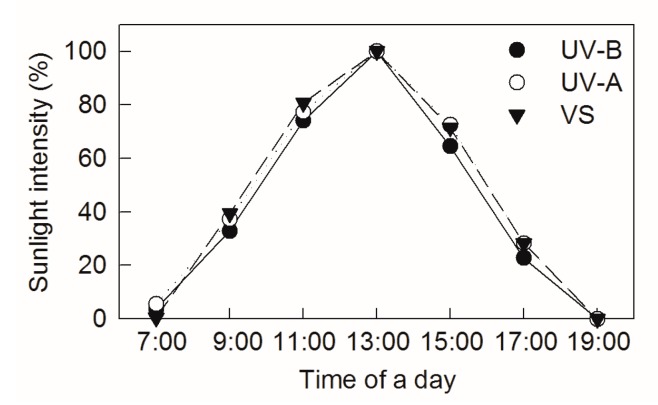
Sunlight intensity on a sunny day. The strongest sunlight intensity at 13:00 was set as 100%. VS represents visible sight.

An external solar electric quantum meter (Spectrum Technologies Company, Chicago, Illinois, USA) and an ultraviolet radiometer (Instrument Factory of Beijing Normal University, Beijing, China) were used to measure visible and ultraviolet light transmittance, respectively. The data were collected at 07:00, 09:00, 11:00, 13:00, 15:00, 17:00, and 19:00 h in an open place on a sunny day. The strongest solar light intensity, recorded at 13:00, was 1800 ± 50 μmolm^−2^ s^−1^ photon flux density and set as 100% (Figure 7). The air temperature and humidity were 28 ± 1 °C and 45%, respectively, at midday during the treatments.

Chemical treatments were carried out in 2017 using ‘Fuji’ fruits. Fruits removed from their bags were sequentially immersed in a 10 μM diphenyleneiodonium chloride (DPI, Sigma-Aldrich, St. Louis, MO, USA, dissolved in 0.2% ethanol) or 0.2% ethanol solution overnight before sunlight exposure. Before sunrise, six groups of apples were placed on wet cotton gauze under diverse layers and meshes of nylon net in an open space for exposure to sunlight, with 20 cm of ground clearance. The relative transmission solar UV-B light intensities were 3.5%, 8.2%, 20.0%, 39.0%, 58.3%, and 73.5%, with shading by 16 layers of 200 mesh screen, 16 layers of 100 mesh screen, 8 layers of 60 mesh screen, 4 layers of 60 mesh screen, 2 layers of 60 mesh screen, or 1 layer of 15 mesh screen, respectively. Other two groups of apples were put into a dark box for dark treatment and on the ground for full exposure, respectively.

Apple peel samples were collected after one week of irradiation. The peels were immediately frozen in liquid nitrogen, then ground to powder and mixed in liquid nitrogen circumstance with an A11 grinder from IKA^®^ Works (VWR, Radnor, PA, USA), and finally stored at −80 °C until further analysis. 

### 4.3. Analysis of Flavonoid Compounds 

The extraction and analysis of flavonoid compounds were carried out as described by Li et al. [29]. Briefly, the frozen tissue powder (0.5 g) was ground in 1.5 mL phenolic compound, extracting a solution containing 70% methanol and 2% formic acid at 0–4 °C. After centrifugation at 10,000 *g* for 20 min, the supernatant was passed through a 0.22-μm syringe filter prior to analysis.

The Inertsil ODS-3 column (5.0 μm particle size, 4.6 mm × 250 mm, GL Sciences Inc., Tokyo, Japan) was used for the separation, preceded by an Inertsil ODS-3 Guard Column. A 10-μL sample of the filtered supernatant was injected into an LC-20A Liquid Chromatograph with a diode array detector (Shimadzu Corporation, Tokyo, Japan). Solvent A consisted of 10% formic acid (11.36% 88% formic acid) in water and solvent B consisted of 10% formic acid and 1.36% water (11.36% 88% formic acid) in acetonitrile. The gradients used were 95% A (0 min), 85% A (25 min), 78% A (42 min), 64% A (60 min), and 95% A (65 min) with a post-run time of 10 min. The flow rate was 1.0 mL min^−1^, and the column temperature was set at 35 °C. Flavonoid compounds were detected at 365 nm for flavonols and 520 nm for anthocyanins.

### 4.4. Analysis of Nucleotide Sugar

The extraction and analysis of nucleotide sugar were carried out as described by Li et al. [29]. Briefly, frozen tissues (0.5 g) were extracted with 1.2 mL of 6% HClO_4_ and 5% insoluble polyvinylpolypyrrolidone (PVPP) at 0–4 °C. After centrifugation at 12,000× *g* for 10 min, 0.8 mL of the supernatant were transferred to another Eppendorf tube and then neutralized with 55 μL of 5 M K_2_CO_3_. The resulting potassium chlorate was removed by 5 min centrifugation at 12,000× *g*. The supernatant was used to measure metabolites. 

The UDP-galactose was separated and quantified using a LC-20A Liquid Chromatograph. The Inertsil ODS-3 column was used in the separation, preceded by an Inertsil ODS-3 Guard Column. A 10-μL sample of the filtered supernatant was injected into the liquid chromatograph. The mobile phase A contained 100 mM KH_2_PO_4_, pH 7.0, and the mobile phase B contained 60% acetonitrile. The gradient for high-performance liquid chromatograph (HPLC) analysis was performed as follows (total 43 min): 100% A (0 min), 100% A (15 min), 90% A (24 min), 0% A (25 min), 0% A (35 min), 100% A (36 min), and 100% A (43 min). The flow rate was changed as follows: 0.3 mL min^−1^ (0 min), 0.3 mL min^−1^ (13 min), 1 mL min^−1^ (15 min), and 1 mL min^−1^ (43 min). The post run-time was 2 min, the column temperature was at 35 °C, and the detection wavelength was 260 nm. 

### 4.5. Quantitative Real-Time Polymerase Chain Reaction (qRT-PCR) Expression Analysis

Total RNA was isolated using the SDS-phenol method according to Malnoy et al. [43]. First-strand cDNA was synthesized using the PrimeScript ^TM^ RT reagent Kit (Takara, Dalian, China), according to the manufacturer’s protocol. All qRT-PCR experiments were performed with the Bio-Rad CFX96 system (Bio-Rad Laboratories, Hercules, CA, USA) with 0.1-mL 8-tube strips, using SYBR Premix Ex TaqTM II (Takara, Dalian, China). *MdActin* was used as the internal reference gene. The PCR amplification program was 95 °C for 3 min, 39 cycles of 95 °C for 10 s, and 57 °C for 30 s, followed by a melting curve analysis program. The primers for *MdActin*, *MdUVR8*, *MdHY5*, *MdCOP1*, *MdMYB10*, *MdCHS*, *MdCHI*, *MdDFR*, *MdANS,* and *MdUFGT* are shown in Table S1.

### 4.6. Analysis of Key Enzyme Activities in Anthocyanin Synthesis

Enzymes were extracted as follows: For CHI (EC 5.5.1.6) and DFR (EC 1.1.1.219), 0.5 g of frozen apple peel tissue were ground in 1.8 mL of 100 mM Tris-HCl buffer (pH 7.5) containing 14 mM β-mercaptoethanol, 5 mM dithiothreitol (DTT), 1% bovine serum albumin (BSA), 0.5% Triton X-100, and 5% PVPP. For CHS (EC 2.3.1.74), 1 g of frozen tissue was homogenized with 5% PVPP in 2 mL of 100 mM sodium phosphate buffer (pH 6.8) containing 14 mM β-mercaptoethanol, 5 mM DTT, 40 mM sodium ascorbate, 3 mM EDTA, 10 μM leupeptin, and 1% BSA at 0–4 °C. For anthocyanin synthase (ANS, EC 1.14.11.19) and UFGT (EC 2.4.1.91), 1 g of frozen tissue was homogenized with 5% PVPP in 2 mL 100 mM Tris-HCl buffer (pH 8.0) containing 14 mM β-mercaptoethanol, 5 mM DTT, 5 mM EDTA, 15 mM MgCl_2_, 0.5% Triton X-100, and 1% BSA. After centrifugation, the enzyme extract was concentrated with ammonium sulfate, as described above. All of the above enzyme extracts were desalted by passage through PD10 columns and were then immediately used for enzyme assays. CHS, CHI, DFR, ANS, and UFGT were assayed as described by Li et al. [29].

### 4.7. Statistical Analysis 

All data were analyzed with *t*-tests (*p* < 0.05) using SPSS 16.0 (SPSS Inc., Chicago, IL, USA). 

## Supplementary Materials

The following are available online at https://www.mdpi.com/1422-0067/20/23/6060/s1.

## Abbreviations

CHS	chalcone synthase
CHI	chalcone isomerase
F3H	flavanone 3-hydroxylase
DFR	dihydroflavonol 4-reductase
ANS	anthocyanidin synthase
UFGT	UDP-glycose: flavonoid 3-O-glycosyltransferase
UVR8	UV RESISTANCE LOCUS 8
COP1	E3 ubiquitin ligase CONSTITUTIVELY PHOTOMORPHOGENIC1
HY5	bZIP transcription factor ELONGATED HYPOGOTYL5
MBW	MYB-bHLH-WD40
MAPKs	mitogen-activated protein kinases
DPI	diphenyleneiodonium chloride
PVPP	polyvinylpolypyrrolidone
BSA	bovine serum albumin
DTT	dithiothreitol
HPLC	high-performance liquid chromatography
qRT-PCR	quantitative real-time polymerase chain reaction
UV-B	ultraviolet-B
ROS	reactive oxygen species
VS	visible sight
