# Supplementary material for: Differential Regulation of Anthocyanin Synthesis in Apple Peel under Different Sunlight Intensities
Source: Int J Mol Sci. 2019 Dec 1;20(23):6060. doi: 10.3390/ijms20236060 (PMC6928825; doi:10.3390/ijms20236060)
Supplement: Supplementary file 1 [file ijms-20-06060-s001.pdf]

Table S1. Primer pairs used for qRT-PCR.

Gene	Accession number	Forward primer (5′ to 3′)	Reverse primer (5′ to 3′)
MdActin	CN938023	TGACCGAATGAGCAAGGAAATTACT	TACTCAGCTTTGGCAATCCACATC
MdUVR8	KU095866	GGAGTGGGTGACAACATC	CGTTCCGATCAACAGATT
MdHY5	AB710143	GTCTTCGAGCTCTGCATTCC	CCTCAACAACCTCTTCAGCC
MdCOP1	AB668569	TGGAAGCTTGTGAGGACTGGGGT	CTCCTCCGATGGCAAATCAAAGA
MdMYB10	DQ267896	TGCCTGGACTCGAGAGGAAGACA	CCTGTTTCCCAAAAGCCTGTGAA
MdCHS	CN944824	GGAGACAACTGGAGAAGGACTGGAA	CGACATTGATACTGGTGTCTTCA
MdCHI	FJ817485	GGGATAACCTCGCGGCCAAA	GCATCCATGCCGGAAGCTACAA
MdDFR	AF117268	GATAGGGTTTGAGTTCAAGTA	TCTCCTCAGCAGCCTCAGTTTTCT
MdANS	AF117269	CCAAGTGAAGCGGGTTGTGCT	CAAAGCAGGCGGACAGGAGTAGC
MdUFGT	AF117267	GCACCGTATGAGCCAAGA	GGGCGTAGAAAAGGAGGAG

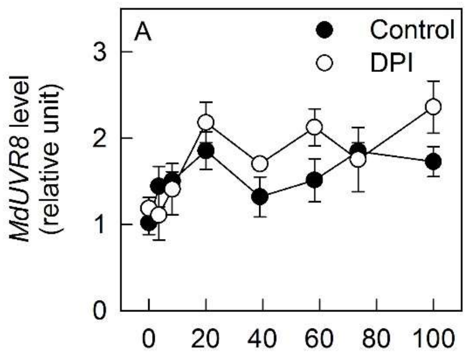

Figure S1. Transcription levels of *MdUVR8* in 'Fuji' apple peels after exposing bagged fruits, with or without DPI treatment, to diverse sunlight intensities. Each data point represents mean ± SE (n = 5). The asterisk indicates a significant difference between DPI treatment and no DPI treatment at $p < 0.05$ (t-test).
